# The overall diet quality in childhood is prospectively associated with the timing of puberty

**DOI:** 10.1007/s00394-020-02425-8

**Published:** 2020-11-02

**Authors:** Ruonan Duan, Tian Qiao, Yue Chen, Mengxue Chen, Hongmei Xue, Xue Zhou, Mingzhe Yang, Yan Liu, Li Zhao, Lars Libuda, Guo Cheng

**Affiliations:** 1 West China School of Public Health and West China Fourth Hospital and Healthy Food Evaluation Research Center, Sichuan University, Chengdu, People’s Republic of China; 2 College of Public Health, Hebei University, Baoding, People’s Republic of China; 3 Department of Clinical Nutrition, Sichuan Academy of Medical Sciences and Sichuan Provincial People’s Hospital, Chengdu, People’s Republic of China; 4 Chinese Academy of Sciences Sichuan Translational Medicine Research Hospital, Chengdu, People’s Republic of China; 5 Nutrition and Health Research Centre, By-Health Co. Ltd, No. 3 Kehui 3rd Street, No. 99 Kexue Avenue Central, Science City, Huangpu District, Guangzhou, People’s Republic of China; 6 Department of Clinical Nutrition, Chengdu First People’s Hospital, Chengdu, People’s Republic of China; 7 Department of Child and Adolescent Psychiatry, University Hospital Essen, University of Duisburg-Essen, Essen, Germany; 8 Laboratory of Molecular Translational Medicine, Center for Translational Medicine, Key Laboratory of Birth Defects and Related Diseases of Women and Children (Sichuan University), Ministry of Education, Department of Pediatrics, West China Second University Hospital, Sichuan University, Chengdu, Sichuan 610041 People’s Republic of China

**Keywords:** Diet quality, Puberty timing, Body composition, Children

## Abstract

**Purpose:**

The influences of nutrition in childhood on puberty onset could have sustained consequences for health and wellbeing later in life. The aim of this study was to investigate the prospective association of diet quality prior to puberty with the timing of puberty onset.

**Methods:**

We considered data from 3983 SCCNG (Southwest China Childhood Nutrition and Growth) study participants with dietary data, anthropometric measurement, and information on potential confounders at their baseline assessment (mean age: 7.1 years for girls and 7.3 years for boys; mean length of follow-up was 4.2 years). Cox proportional hazard regression estimating hazard ratios (HRs) and 95% confidence intervals (CIs) were used to examine the relationship between diet quality and puberty onset. Dietary intake at baseline was assessed using a validated food frequency questionnaire. Diet quality was determined using the Chinese Children Dietary Index (CCDI) which measures adherence to current dietary recommendations (theoretical range: 0–160 points). Age at Tanner stage 2 for breast/genital development (B2/G2), menarche or voice break (M/VB) were used as pubertal markers.

**Results:**

The CCDI score ranged from 56.2 to 136.3 for girls and 46.1–131.5 for boys. Pubertal markers consistently indicate that girls and boys with higher diet quality were more likely to enter their puberty later than their counterparts with lower CCDI scores (higher vs. lower CCDI tertiles: adjusted HR for age at B2: 0.85 (95% CI, 0.81–0.94), *p* for trend = 0.02; G2: 0.86 (95% CI,0.80–0.96), *p* for trend = 0.02; M: 0.86 (95% CI,0.80–0.95), *p* for trend = 0.02; VB: 0.86 (95% CI,0.79–0.98), *p* for trend = 0.03), after adjustment for paternal education level, baseline energy intake, and pre-pubertal body fat.

**Conclusions:**

Our data suggested a later puberty onset and later timing of progressed puberty stages in children with a high diet quality, which were independent of pre-pubertal body fat.

**Electronic supplementary material:**

The online version of this article (10.1007/s00394-020-02425-8) contains supplementary material, which is available to authorized users.

## Introduction

Age of puberty onset is considered to be of general public health relevance. An early age at puberty onset may be an intermediary factor on the life-course path to a number of hormone-related cancers (breast [1], ovarian [2], endometrial [3], prostate [4, 5] and testicular cancer [6]), all-cause mortality [7], and has also been linked to later insulin resistance [8] and adiposity [9]. In this context, factors influencing puberty timing are increasingly acknowledged.

To date, the influence of modifiable risk factors such as nutrition on puberty onset has been addressed: some [10–12], but not all [13–15] prospective observational studies suggested a role for dietary intakes of nutrients or food groups during pre-puberty: higher energy intake [16] and intakes of animal protein [10, 12], milk [17, 18], red meat [11], and sweetened soft drinks [19, 20] were associated with earlier menarche, whereas higher intakes of total protein [21], carbohydrate [16], fiber [22], isoflavones [23], or yogurt [24] were related to later menarche. However, most of these studies [10–15, 17–24] were focused solely on a single pubertal marker. The use of different pubertal markers covering the range from earlier [pubertal stage 2 for breast in girls/genital development in boys (B2/G2)] to later stages [menarche in girls or voice break in boys (M/VB)] of pubertal development might help to elucidate potential mechanisms involved. Moreover, most of the above studies [10–15, 17–24] were conducted in Western countries. In recent years, a secular trend of earlier puberty onset has been observed in both Chinese boys [25] and girls [26]. Few studies have been addressed the influence of dietary factors on puberty timing among Chinese children.

In addition, previous studies considered only single or a few nutrients or foods. It is conceivable that nutrients, food groups, and/or eating behaviours may interact or have additive effects on puberty onset which might be best described when total diet quality is assessed. Dietary indices [27], which measure the extent to which current dietary recommendations are met, can thus provide additional insight into the effect of diet as a whole. To date, two prospective observational studies suggested that US girls with higher adherence to Mediterranean-like diet [28] as well as German children with higher overall diet quality according to German dietary recommendation [29] reached their puberty onset later. To date, one dietary index [30] has been developed for Chinese children, Chinese Children Dietary Index (CCDI), which measures adherence to current dietary recommendations [31] and could, thus, be used to analyze associations between pre-pubertal diet quality and timing of puberty in Chinese children.

The current obesity epidemic has received special attention since body composition in childhood may potentially influence the timing of puberty [32]. In recent years, the prevalence of childhood obesity has risen alarmingly in China [33]. Hence, it is important to examine whether a potential impact of the overall diet quality on the puberty timing is affected by pre-pubertal body composition.

Using prospectively collected data from the Southwest China Childhood Nutrition and Growth (SCCNG) Study, we thus investigated whether the extent to which current dietary recommendations are met in the years preceding puberty onset was associated with the timing of early (B2/G2) and late (M/VB) pubertal markers. Furthermore, we tested whether this association was affected by pre-pubertal body composition.

## Subjects and methods

### Study sample

The recruitment of SCCNG study participants started in March 2013 in Sichuan Province, Guizhou Province and Chongqing Municipality, which are located in Southwest China. Details on the subject selection procedure and the study protocol have been described elsewhere [34]. In brief, for the yearly recruitment from 2013 onwards eligible participants were children aged 6–8 years in 23 selected primary schools who are cooperative and voluntary at the time of recruitment. At the first examination, information on socio-demographic issues, dietary intake and eating behaviours, physical activity and sedentary behaviours, anthropometry and pubertal development is obtained. From then on, detailed data on nutrition, growth, metabolism, and health status are collected at regular intervals until the age of 15: the assessments of anthropometry and puberty status are conducted every year, while data of dietary intake and physical activity are collected biennially. The study was approved by the Ethics Committee of the Sichuan University, and all examinations and questionnaires were performed with parental consent.

**Fig. 1:**
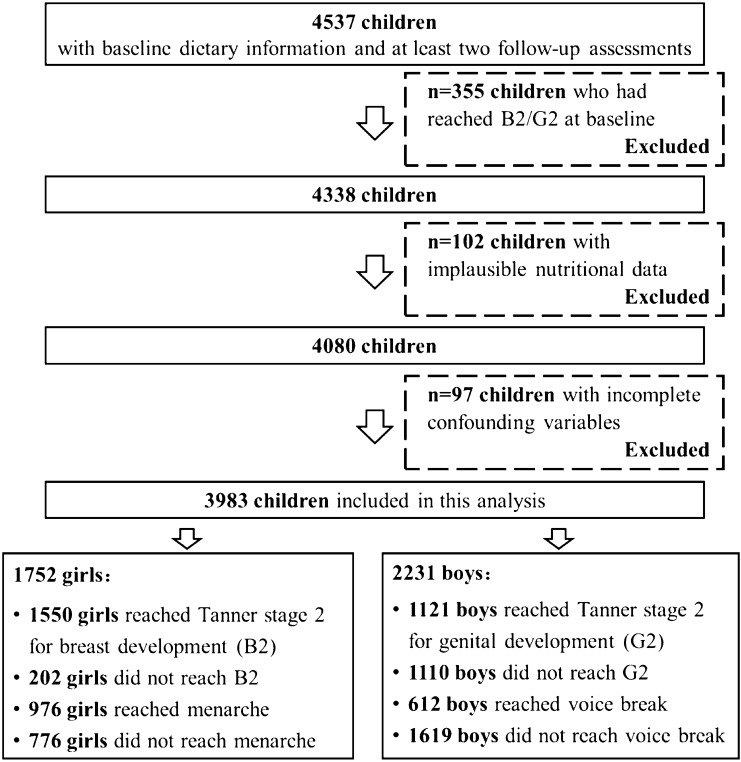
Flowchart for the study sample

Between January 2013 and December 2018, 6967 children aged 6–8 years were included for baseline. Of these, 4537 children had completed at least 2 follow-up assessments by the end of 2019. Since we were interested in the prospective relevance of diet quality on puberty timing, 355 children who had already reached B2/G2 at baseline were excluded. 102 participants with implausible energy intakes at baseline according to the age- and sex-specific cut-offs [35] and 97 participants with incomplete information on potential confounders were further excluded. Finally, 3983 children (1752 girls and 2231 boys) were eligible for the present analysis (Fig. 1).

### Nutrition assessment and eating behaviors

The present analyses based primarily on the nutrition data collected via a validated food frequency questionnaire (FFQ) [36] for the consumption of foods and food groups over the past year. Trained investigators collected the information on dietary intake of children from their caregivers in the face-to-face interview. The FFQ included 53 foods or food groups that were most representative of local consumption grouped into 17 categories (whole grains, refined grains, tubers, vegetables, fruits, nuts, meat, fish and shrimp, animal viscera, eggs, dairy and dairy products, soybeans and its products, fried foods, sugary snacks, sugar-sweetened beverages, fruit juice and dietary supplements). Parents were asked to report their children’s frequency (never, daily, weekly, monthly and annually) for each food item and the estimated portion size. To increase accuracy in portion size estimation, food models and picture aids were provided. During the interview, the investigators checked FFQs for potentially incorrect responses and clarified with participants’ caregivers when necessary. Dietary intake data are converted into energy and nutrient intake data using the continuously updated in-house nutrient database based on NCCW software (version 11.0, 2014), which reflects the China Food Composition.

In addition, 3-day 24-h recalls were used in the SCCNG to collect dietary intake data by trained investigators. For children younger than 9 years, their caregivers provided the information on food consumption at home, while children provided the dietary intake information from school themselves. Details on recipes and brands of all food items reported were inquired. Food models, standard tableware including bowls, plates and glasses were provided to improve the accuracy of the portion-size estimates. In addition, children were given a photo book, which contains photos of snacks and beverages and the pictures of the commonly used commercial packaging (e.g., one carton) to improve the diet recall. For this study, dietary intake data from 24-h recalls was used in our sensitivity analysis.

For eating behaviors relevant to diet quality of children, participants were asked whether they often ate breakfast (at least 5 days per week) or had dinner with parents/grandparents regularly (at least 5 days per week).

### Measurement of dietary quality

The Chinese Children Dietary Index (CCDI) [30] was initially developed to rate diet quality in children by scoring their nutritional intakes in relation to the Chinese dietary intake recommendations (Chinese Dietary Guidelines 2007 [37] and Chinese Dietary Reference Intakes 2013 [38]) and addressing health-promoting behaviours. Modified CCDI, which measures adherence to current Chinese dietary recommendation, was used in the present analysis. The modified CCDI was based on the Chinese Dietary Guidelines 2016 [31], which provided specific dietary intake recommendations for children aged 7–10 years, 11–13 years, and 14–17 years, respectively. The development of modified CCDI and its calculation are described in detail elsewhere [39]. Briefly, the CCDI incorporated 16 components, including grains, vegetables, fruits, dairy and dairy products, soybeans and its products, meat, fish and shrimp, eggs, drinking water, sugar-sweetened beverages, vitamin A, fatty acids, fiber, dietary variety, eat breakfast or have dinner with parents/grandparents regularly, and energy balance. Each component in CCDI contributed 0–10 points to the total score and children who consumed appropriate types and amounts of foods or nutrients and engaged in health-promoting behaviors received full points for each component. Therefore, the range of CCDI score is 0–160, with a higher score indicating a better diet quality.

### Assessment of puberty onset

In the SCCNG study, pubertal maturation for breast (girls) and pubic hair (girls and boys) stages were assessed at each examination by investigators according to the standardized criteria of Tanner stages [40]. Testicular volume was assessed by comparative palpation with the Prader orchidometer. If the testicular volumes of the two testes were not equal, the volume of the larger one was recorded. Testicular volume less than 1 mL was recorded as 1 mL. In addition, during the annual physical examination for children, children were asked whether menarche (girls) or voice break (boys) had already occurred and the respective month and year were recorded.

For this analysis, age at Tanner stage 2 for breast development in girls and the initiation of gonadal growth in boys as well as age at menarche in girls and age at voice break in boys, respectively, were considered.

### Anthropometry

An Ultrasonic Weight and Height Instrument (DHM-30, Dingheng Ltd, Zhengzhou, China) was used to assess standing height to the nearest 0.1 cm and weight to the nearest 0.1 kg with the subjects dressed lightly and barefoot. Additionally, skinfold thicknesses of triceps and subscapular angle sites were measured on the right side of the body to the nearest 0.1 mm using a Holtain caliper (Holtain Ltd, Crymych, UK). All anthropometric measurements were performed twice and the averages were calculated for each child.

For body mass index (BMI) sex- and age-independent BMI SDS were calculated using the Chinese reference curves [41]. Overweight was defined according to the International Obesity Task Force (IOTF) BMI cut-offs for children, which correspond to an adult BMI of 25 kg/m^2^ [42]. Percent body fat (%BF) was calculated using the Slaughter equations [43].

### Covariates

Parents were asked to provide information about pregnancy and infancy (i.e., children’s birth weight, exclusive breastfeeding duration, timing of complementary feeding), and family characteristics (i.e., place of residence, household income, family size, smoking in the household, parental age, parental occupation and education levels).

A validated physical activity questionnaire (PAQ) was used to collect information on the frequency, duration and type of physical activity inside and outside school settings as well as leisure-time activities. The PAQ included a checklist of 38 items, i.e., walking, running, climbing stairs, ball games, dancing and so on. In addition, participants were asked to report the usual time spent on sedentary behaviours, i.e., watching television, using computers, using smart phones and doing homework) on weekdays and on weekends.

### Statistical analysis

SAS^®^ procedures (version 9.4, SAS Inc, Cary, NC) were used for all data analyses. All analyses were performed with significance level at *p* < 0.05.

To obtain three categories characterizing a lower, moderate and higher diet quality, the continuous CCDI scores were divided into three groups based on the sex-specific tertiles. Kolmogorov–Smirnov and Shapiro–Wilk tests were conducted to test the normality of the data. Birth weight and percent body fatness at baseline were not normally distributed and presented as medians (25th percentile, 75th percentile), while other continuous variables were normally distributed and presented as means (SD). Differences in anthropometric data, socio-demographic data and nutritional intake between groups of CCDI were tested using ANOVA test for normally distributed continuous variables, Kruskal–Wallis test for not normally distributed continuous variables, followed by Student–Newman–Keuls tests or Dunn’s post hoc tests. Chi square test was used for categorical variables. Since different pubertal markers were used in boys and girls, statistical models as well as descriptive tables were stratified by sex.

To investigate the prospective relevance of diet quality at baseline according to CCDI with age at B2/G2 and M/VB, Cox proportional hazard regression models were used. Censoring occurred at the age of reaching B2/G2, M/VB or age at last follow-up assessment if the respective pubertal stage had not been achieved. In the basic models, the tertiles of CCDI score at baseline were the principle independent fixed effects. Cox regression models considered birth weight, age at baseline, physical activity, family income, paternal/maternal educational level, smoking in the household, and total energy intake at baseline as potential confounders. Each potential confounder was initially considered separately and included if it was associated with both the dietary index and indicators of puberty timing and substantially altered the estimate by more than 10% in both girls and boys. Thus, paternal education level and energy intake at baseline were adjusted for in model 2. In a final model, percent body fatness at baseline was additionally included because it has been proposed that childhood body composition might be related to puberty timing [44]. Hazard ratios (HRs) and 95% confidence intervals (CIs) were estimated by comparing the moderate and higher diet quality to the lower diet quality in these models.

To test the robustness of our results, CCDI scores were recalculated using dietary data estimated from three 24-h recalls instead of FFQ data and included in the models.

## Results

In total, 3983 children (1752 girls and 2231 boys) were included in the present analyses. The mean age at baseline was 7.1 (0.7) years for girls and 7.3 (0.8) years for boys. They were followed up 4.2 years. Among girls, 1550 (88.5%) had reached B2, and 976 (55.7%) had experienced menarche. Among boys, 1121 (50.2%) had reached G2, and 612 (27.4%) had experienced voice break. There were no significant differences in age at baseline, or the BMI SDS or percentage body fat between these 3983 children and the 102 children who provided pubertal data and anthropometry, but were excluded from this analysis because of missing or implausible nutritional data (data not shown).

In our participants, the CCDI score estimated with the individual mean values of dietary data at baseline ranged from 56.2 to 136.3 for girls and 46.1–131.5 for boys (Table 1). Generally, the diet quality of girls (CCDI score: 90.7 (15.5)) was higher than that of boys (CCDI score: 87.9 (14.9)) (*p* < 0.0001). Boys and girls with a higher diet quality according to the CCDI were more likely to have a higher educated father and to live in a household with less smoking compared to their counterparts with lower diet quality. Girls with a higher diet quality consumed significantly more carbohydrates and less protein and fat, while boys with a higher diet quality consumed significantly more carbohydrate and less protein. Despite the fact that the components of the CCDI partly account for energy intake, a higher diet quality tended to be associated with a lower energy intake (*p* = 0.06). In both genders, a higher diet quality was associated with a lower %BF, a later age at Tanner stage B2 and menarche for girls and a later age at voice break for boys as compared to their counterparts with a lower diet quality.

**Table 1:** Characteristics of participants by group of diet quality^a^ at baseline

	Diet quality according to CCDI score			*p*
	Lower (56.2–82.3)^b^	Moderate (82.9–105.6)^b^	Higher (106.2–136.3)^b^	
Girls (*n* = 1752)				
* N* (%)	584 (33.3)	584 (33.3)	584 (33.3)	
Birth weight (kg)	3.1 (2.8, 3.8)	3.3 (2.9, 3.7)	3.2 (2.7, 3.8)	0.3
Age at baseline (years)	7.1 (0.8)	7.3 (0.8)	7.2 (0.9)	0.1
Age at menarche (years, *n* = 976)	12.6 (0.8)	12.8 (0.9)*	13.2 (1.0)#※	0.02
Age at Tanner stage B2^d^ (years, *n* = 1550)	9.0 (1.4)	9.3 (1.5)*	9.6 (1.2)#※	0.01
BMI SDS at baseline (kg/m^2^)	0.3 (0.8)	0.2 (0.7)	0.2 (0.8)	0.1
Percent body fatness^e^ at baseline (%)	16.9 (15.3, 19.6)	16.5 (15.1, 18.4)*	15.7 (13.6, 18.8)#※	0.04
Overweight^f^ (*n* (%))	77 (13.2)	66 (11.3)	64 (11.0)	0.1
High physical activity (*n* (%))	153 (26.2)	168 (28.8)	166 (28.4)	0.06
Duration of breastfeeding (weeks)	16.8 (5.9)	15.7 (5.3)	18.6 (6.3)	0.07
Parental data at baseline				
High family income^g^ (*n* (%))	122 (20.8)	130 (22.3)	137 (23.5)	0.06
High paternal educational level^h^ (*n* (%))	117 (20.0)	137 (23.5)*	151 (25.9)#※	0.04
High maternal educational level^h^ (*n* (%))	105 (18.0)	108 (18.5)	111 (19.0)	0.07
Smoking in the household (*n* (%))	386 (66.1)	328 (56.2)*	284 (48.6)#※	0.02
Mother’s age at menarche (years)	12.0 (0.9)	12.3 (0.8)*	12.5 (1.1)#※	0.05
Nutritional data^i^				
Total energy intake (kcal/d)	1401 (228)	1626 (237)	1531 (232)	0.06
Carbohydrates (% of energy)	53.1 (5.2)	58.8 (4.3)*	57.3 (4.5)#※	0.04
Fat (% of energy)	32.3 (4.9)	26.1 (4.3)*	29.0 (4.4)#※	0.02
Protein (% of energy)	14.6 (2.2)	15.1 (2.1)*	13.7 (1.9)#※	0.03

	Lower (46.1–77.5)^j^	Moderate (78.2–101.7)^j^	Higher (102.3–131.5)^j^	*p*
Boys				
* N* (%)	743 (33.3)	744 (33.3)	744 (33.3)	
Birth weight (kg)	3.6 (3.1, 3.9)	3.3 (2.8, 4.1)	3.4 (2.8, 3.9)	0.1
Age at baseline (years)	7.0 (0.9)	7.3 (0.7)	7.1 (0.8)	0.1
Age at voice break (years, *n* = 612)	13.5 (1.2)	13.8 (1.3)*	14.1 (1.1)#※	0.03
Age at pubertal stage G2^d^ (years, *n* = 1121)	10.9 (1.2)	11.2 (1.4)	11.4 (1.0)	0.05
BMI SDS at baseline (kg/m^2^)	0.3 (0.8)	0.3 (0.6)	0.2 (0.7)	0.3
Percent body fatness^e^ at baseline (%)	13.9 (11.4, 17.5)	13.6 (10.2, 17.3)*	13.5 (11.0, 17.8)#※	0.04
Overweight^f^ at baseline (*n* (%))	104 (14.0)	89 (12.0)	83 (11.2)	0.09
High physical activity (*n* (%))	197 (26.5)	219 (29.4)	215 (28.9)	0.06
Duration of breastfeeding (weeks)	19.2 (5.2)	20.5 (5.8)	20.6 (6.1)	0.08
Parental data at baseline				
High family income^g^ (*n* (%))	152 (20.5)	153 (20.6)	158 (21.2)	0.07
High paternal educational level^h^ (*n* (%))	166 (22.3)	176 (23.6)*	187 (25.1)#※	0.03
High maternal educational level^h^ (*n* (%))	135 (18.2)	136 (18.3)	141 (19.0)	0.05
Smoking in the household (*n* (%))	499 (67.2)	425 (57.1)*	391 (52.6)#※	0.03
Mother’s age at menarche (years)	12.2 (0.7)	12.4 (0.9)	12.3 (0.8)	0.08
Nutritional data^i^				
Total energy intake (kcal/d)	1910 (237)	1816 (218)	1772 (223)	0.06
Carbohydrates (% of energy)	58.7 (6.5)	59.8 (6.2)*	61.0 (5.8)#※	0.03
Fat (% of energy)	26.4 (4.6)	27.3 (4.2)	26.5 (4.4)	0.05
Protein (% of energy)	14.9 (2.4)	12.9 (2.2)*	12.5 (2.0)#※	0.04

^a^Values are means (SD), medians (Q1, Q3) or frequency; CCDI, Chinese Children Dietary Index, based on dietary data at baseline

^b^Values are min–max in tertiles in girls

^c^Test for difference between the groups was performed, using ANOVA test for normally distributed continuous variables, Kruskal–Wallis test for not normally distributed continuous variables, followed by Student–Newman–Keuls tests or Dunn’s post hoc tests, and Chi square test for categorical variables. *P < 0.05 between lower and moderate diet quality group, #*P* < 0.05 between moderate and higher diet quality group < 0.05, ※*P* < 0.05 between lower and higher diet quality group

^d^Tanner stage 2 for breast development (girls) or the initiation of gonadal growth (boys)

^e^Calculated according to Slaughter et al. [43]

^f^Definition according to the International Obesity Task Force (IOTF) [53]

^g^Average annual income of family at least ≥ 35,000 CNY (Chinese Yuan), which is moderate level among the general population in South China

^h^School education at least 12 years

^i^Mean values of dietary data at baseline using food frequency questionnaires

^j^Values are min–max in tertiles in boys

The dietary intake of each component of CCDI estimated with individual mean values of dietary data at baseline and sub-scores of CCDI are presented in Table 2. Among girls, the highest median sub-scores, i.e., adherence to dietary recommendations, were observed for fatty acids, breakfast and dinner, energy balance component, and dairy and dairy products (10.0 for fatty acids, 9.0 for breakfast and dinner, 8.3 for energy balance, 8.0 for dairy and dairy products). However, scores for eggs and fish and shrimp were much lower (≤ 3 points), reflecting excessive meat consumption and inadequate consumption of fish, shrimp and eggs. More than 60% of our girls consumed vegetables and fruits below the recommendations issued by Chinese Dietary Guidelines resulting in low mean sub-scores especially for vegetables (4.6) and also for dietary fiber (3.9).

**Table 2:** Dietary intake^a^ by groups of diet quality and the medians (25th percentile, 75th percentile) of CCDI sub-scores^b^

	Diet quality according to CCDI score			Sub-scores of CCDI	Percentage of participants meeting dietary recommendations
	Lower (56.2–82.3)^c^	Moderate (82.9–105.6)^c^	Higher (106.2–136.3)^c^		
Girls					
Grains, g/d	487 (402, 589)	346 (263, 379)	172 (115, 263)	5.7 (3.3, 7.8)	12.6%
Vegetables, g/d	99 (81, 123)	159 (136, 178)	263 (252, 296)	4.6 (2.9, 7.4)	16.1%
Fruits, g/d	41 (29, 62)	115 (97, 136)	163 (148, 207)	7.0 (3.6, 10.0)	37.8%
Dairy and dairy products, g/d	67 (54, 95)	243 (201, 252)	294 (279, 345)	8.0 (3.5, 10.0)	38.1%
Soybeans and its products, g/d	3.6 (0, 7.1)	13 (8.9, 17)	57 (39, 89)	4.5 (2.4, 10.0)	26.7%
Meats, g/d	73 (11, 136)	65 (33, 102)	41 (35, 82)	3.3 (0, 7.1)	14.7%
Fishes and shrimps, g/d	0 (0, 0)	6 (3, 19)	25 (19, 38)	0 (0, 5.1)	9.3%
Eggs, g/d	0 (0, 0)	15 (10, 25)	58 (43, 86)	2.7 (0, 8.0)	13.1%
Drinking water, mL/d	350 (225, 425)	575 (500, 750)	975 (925, 1200)	5.0 (3.3, 8.3)	17.2%
SSBs^d^, mL/d	155 (128, 201)	78 (35, 103)	0 (0, 0)	4.5 (2.2, 10.0)	26.9%
Vitamin A, μgRE/d	181 (160, 212)	325 (293, 346)	465 (428, 492)	5.5 (3.2, 8.9)	21.2%
Fatty acids^e^	1.6 (1.2, 2.0)	2.4 (2.0, 2.9)	3.4 (3.0, 3.8)	10.0 (3.9, 10.0)	55.1%
Dietary fiber, g/d	5.2 (4.3, 6.5)	8.5 (7.2, 9.7)	12 (9.8, 14)	3.9 (2.4, 4.5)	11.7%
Diet variety (servings)^f^	6 (4, 9)	9 (6, 13)	12 (9, 16)	7.0 (4.0, 9.0)	21.2%
Breakfast and dinner^g^	5 (4, 8)	7 (5, 10)	9 (8, 11)	9.0 (7.0, 10.0)	39.1%
Energy balance^h^	0.7 (0.4, 2.2)	0.9 (0.6, 1.6)	1.1 (0.7, 1.3)	8.3 (3.5, 9.9)	23.9%

	Lower (46.1–77.5)^i^	Moderate (78.2–101.7)^i^	Higher (102.3–131.5)^i^	Distribution of sub-scores	Percentage of participants meeting dietary recommendations
Boys					
Grains, g/d	535 (489, 553)	447 (311, 514)	215 (162, 348)	2.7 (0, 6.1)	9.1%
Vegetables, g/d	87 (71, 112)	145 (121, 163)	238 (218, 251)	4.5 (2.4, 6.9)	11.2%
Fruits, g/d	26 (19, 39)	141 (112, 175)	178 (165, 213)	6.5 (1.2, 10.0)	31.6%
Dairy and dairy products, g/d	81 (69, 96)	275 (230, 319)	348 (316, 391)	8.6 (5.4, 10.0)	41.3%
Soybeans and its products, g/d	0 (0, 0)	12 (7.4, 23)	73 (41, 102)	3.8 (0, 10.0)	25.2%
Meats, g/d	98 (65, 187)	78 (41, 127)	46 (35, 79)	1.2 (0, 5.9)	8.3%
Fishes and shrimps, g/d	0 (0, 0)	7 (2, 19)	31 (21, 45)	0 (0, 7.0)	7.2%
Eggs, g/d	0 (0, 0)	24 (16, 39)	73 (52, 105)	3.4 (0, 10)	25.8%
Drinking water, mL/d	425 (325, 475)	650 (525, 800)	1075 (875, 1200)	5.7 (4.0, 8.9)	19.8%
SSBs^d^, mL/d	182 (119, 237)	85 (48, 117)	0 (0, 0)	4.0 (2.3, 10.0)	25.5%
Vitamin A, μgRE/d	166 (151, 182)	293 (270, 338)	442 (408, 486)	5.1 (3.0, 8.9)	19.5%
Fatty acids^e^	1.5 (1.1, 1.8)	2.4 (2.0, 2.9)	3.7 (3.3, 3.8)	10.0 (5.0, 10.0)	58.1%
Dietary fiber, g/d	4.2 (3.1, 5.5)	7.3 (6.1, 8.7)	10 (9.0, 13)	3.6 (2.1, 4.5)	9.4%
Diet variety^f^	6 (4, 9)	8 (7, 11)	10 (8, 14)	6.0 (4.0, 8.0)	19.6%
Breakfast and dinner^g^	6 (5, 9)	8 (6, 10)	9 (7, 11)	8.0 (6.0, 10.0)	29.1%
Energy balance^h^	1.2 (0.5, 2.6)	0.8 (0.5, 1.2)	1.1 (0.7, 1.3)	8.1 (3.0, 9.7)	23.0%

^a^All nutritional data represent crude values per day. Mean values of dietary data at baseline

^b^Data are presented as medians (25th percentile, 75th percentile) in tertiles, *n* = 3983. *CCDI* Chinese Children Dietary Index, based on dietary data at baseline

^c^Values are min–max in tertiles in girls

^d^SSBs, sugar-sweetened beverages. SSBs were defined as beverages with added sugar, such as lemonades, fruit drinks (diluted and sugar-sweetened fruit juices), ice teas and so on. Juices made from 100% fruit were not classified as SSBs

^e^Ratio of poly- and monounsaturated fatty acids to saturated fatty acids, derived from the HEI-2010 [54]

^f^Daily consumption of at least one serving from each of the food groups (grains, vegetables, fruits, dairy/beans and meats/poultry/fishes/eggs) was necessary to count the diet variety

^g^Frequencies per week, having breakfast and dinner with parents or grandparents

^h^Energy balance was reflected by energy intake and time spent on sedentary behaviors. Energy expenditure was reflected by time spent on sedentary behaviors, i.e., watching television, using computers and doing homework

^i^Values are min–max in tertiles in boys

Among boys, the overall distribution of the CCDI components was comparable, with high mean sub-scores observed for fatty acids and dairy and dairy products, and low scores for fish and shrimp, and meat. In contrast to girls, the sub-score for grains was lower than 3 points, reflecting a diet with excessive grain and meat consumption and inadequate consumption of fish and shrimps in boys. More than 75% of our boys consumed vegetables and soybeans and its products below the recommendations issued by Chinese Dietary Guidelines; the mean sub-scores for vegetables, soybeans and its products and dietary fiber were 4.5, 3.8 and 3.6, respectively. Similar distributions of each component of CCDI in girls and boys were found when using dietary values estimated from 24-h recalls (Table S1).

Cox proportional hazard regression models revealed positive associations of diet quality at baseline with early and late markers of puberty (Table 3) in both genders, which remained significant when the potential confounder body fat at baseline was included in the final model. According to the final models, girls with a higher diet quality were more likely to reach their B2 [adjusted HR: 0.85 (95% CI, 0.81–0.94), *p* for trend = 0.02] and menarche [adjusted HR: 0.86 (95% CI, 0.80–0.95), *p* for trend = 0.02] later than girls with a lower diet quality. Similarly, boys with a higher diet quality were more likely to experience their G2 [adjusted HR: 0.86 (95% CI, 0.80–0.96), *p* for trend = 0.02] and VB [adjusted HR:0.86 (95% CI, 0.79–0.98), *p* for trend = 0.03] than boys with a lower diet quality at a later age.

**Table 3:** Association of diet quality in childhood with puberty timing^a^

	Diet quality according to CCDI score			*p*_*trend*_^c^
	Lower (56.2–82.3)^b^	Moderate (82.9–105.6)^b^	Higher (106.2–136.3)^b^	
Girls				
Age at Tanner stage B2 (*n* = 1752)				
Unadjusted model:	1	0.90 (0.81, 0.97)	0.86 (0.79, 0.93)	0.04
Model 2^d^:	1	0.88 (0.78, 0.96)	0.85 (0.80, 0.92)	0.02
Final model^e^:	1	0.88 (0.79, 0.98)	0.85 (0.81, 0.94)	0.02
Age at menarche (*n* = 1752)				
Unadjusted model:	1	0.91 (0.83, 0.98)	0.88 (0.81, 0.94)	0.03
Model 2^d^:	1	0.89 (0.81, 0.97)	0.87 (0.79, 0.95)	0.02
Final model^e^:	1	0.89 (0.80, 0.97)	0.86 (0.80, 0.95)	0.02

	Lower (46.1–77.5)^f^	Moderate (78.2–101.7)^f^	Higher (102.3–131.5)^f^	*p*_*trend*_^c^
Boys				
Age at Tanner stage G2 (*n* = 2231)				
Unadjusted model:	1	0.92 (0.84, 1.02)	0.87 (0.78, 0.95)	0.03
Model 2^d^:	1	0.90 (0.82, 0.98)	0.86 (0.79, 0.96)	0.02
Final model^e^:	1	0.89 (0.82, 0.97)	0.86 (0.80, 0.96)	0.02
Age at voice break (*n* = 2231)				
Unadjusted model:	1	0.93 (0.86, 1.05)	0.85 (0.74, 1.04)	0.05
Model 2^d^:	1	0.91 (0.85, 1.01)	0.86 (0.76, 0.97)	0.04
Final model^e^:	1	0.90 (0.83, 0.98)	0.86 (0.79, 0.98)	0.03

^a^Values are models adjusted hazard ratios (95% CI), *HR* = hazard ratio; diet quality was assessed according to CCDI (Chinese Children Dietary Index) based on diet data at baseline

^b^Values are min–max in tertiles in girls

^c^*P* for trend across CCDI tertiles were performed by including CCDI ordinals as continuous variables

^d^Adjusted for paternal education level and energy intake at baseline

^e^Additionally adjusted for percent body fat at baseline

^f^Values are min–max in tertiles in boys

Our results were similar in sensitivity analyses. When we used dietary values estimated from 24-h recalls to examine associations between diet quality at baseline and puberty timing, relation estimates were similar (Table S2).

## Discussion

Our study suggests that both girls and boys who had a higher diet quality in pre-puberty, as indicated by the CCDI, experienced their puberty timing at a later age than children with a lower diet quality. This association was observed independently of their pre-pubertal body fat.

Puberty onset is a milestone of growth for both boys and girls. An earlier puberty onset could represent one of the immediate health outcomes associated with diet quality in childhood. In 2010, data from 222 Dortmund Nutritional and Anthropometric Longitudinally Designed (DONALD) study participants showed that children with higher diet quality entered puberty approximately 0.4 year later than their counterparts with lower diet quality [29]. Based on the working experience of the DONALD Study, we designed the SCCNG with large sample size and detailed assessments of dietary intake as well as frequent follow-ups to investigate the prospective relevance of diet quality on growth development among Chinese children. The difference in early and late puberty markers between children with a lower diet quality and children with a higher diet quality observed in our study may have long-term consequences for risk of cancers including breast [1], ovarian [2], endometrial [3], prostate [4, 5] and testicular cancer [6]. In this analysis, the diet quality was examined at 4.2 years preceding puberty onset, which suggested that the diet quality in childhood may have potentially long-term effects for puberty timing. Moreover, the influence of higher diet quality on later puberty timing was found both in girls and boys in the present study. A meta-analysis of 12 cohort studies with 2,341,769 participants has demonstrated that a one year delay in menarche is associated with a 3.3% lower all-cause mortality or 4.1% lower ischemic heart disease mortality [7]. Although such data are only available for menarche, we believe that our finding of 14–15% lower probability of experiencing puberty timing at earlier age in those with a higher diet quality may thus be of public health relevance. In addition, our results have a more direct public health implication than studies focusing on single nutrients: a closer adherence to current dietary recommendation appears to yield a later puberty onset. In the present study, we could use the dietary index which is currently available specifically for Chinese children. In view of the increasing trend towards earlier puberty onset among Chinese children, public health initiatives should be tailored at improving the quality of dietary intake.

In this study, both boys and girls with a higher diet quality according to CCDI were more likely to have higher intakes of dairy and dairy products, dietary fiber, vegetables, fruits and soybeans, lower intakes of meat and SSB, and having breakfast and dinner more with parents and grandparents. Our findings are thus in line with previous prospective observational studies focusing on single nutrients and food groups, which suggested that higher intakes of fiber [22] or isoflavones [23], or lower meat [11, 12] or SSB [19, 20] consumptions were associated with a later timing of menarche. Previous studies have suggest that diet rich in fiber and isoflavones may delay timing of puberty by increasing sex hormone-binding globulin and decreasing endogenous estrogens [28, 45] among girls. Conversely, intake of SSB may contribute to high levels of insulin secretion and insulin-like growth factor1 (IGF-1), which could result in early onset of puberty [19, 46]. The robust association in our study may hence reflect a combined effect of these foods and food groups. However, the underlying mechanisms of the impact of dietary intake on boys’ puberty timing require further research. Interestingly, additional analyses suggested that the association of the CCDI with the puberty timing was partly driven by eating behaviors: having breakfast and dinner more with parents and grandparents, especially among girls (data not shown). This might suggest that the health promoted eating behaviors may be crucial with respect to puberty timing [47]. Hence, public health initiatives should not be only concentrated in improving the consumption of nutrients or foods/food groups, but also focused on the health promoted eating behaviors or eating environment.

In this study, the association of diet quality with puberty timing was not affected by body composition in pre-puberty, although our children with a lower diet quality seem to have more body fat. Instead, higher fat intakes and lower intakes of vegetables and fiber [48] among those with a lower diet quality may have enhanced the availability of circulating estrogen or be associated with higher leptin concentrations, thereby influencing pubertal development [49]. Thus, the association of diet quality in pre-puberty with the timing of puberty onset is independent of the pre-pubertal body composition, but may have been mediated by estrogen and leptin metabolism.

Our study has several strengths. In contrast to previous studies focused only on girls, this study examined data from both girls and boys. The prospective nature, and repeated detailed measurements of anthropometric, pubertal and dietary data in participants in conjunction with the ability to adjust for a number of major potential confounders both in children and in their parents were the considerable strengths. Moreover, our participants and their parents/family were representative of the general population in age, economic and education status according to the regional statistic books [50–52].

Some limitations of our study should be mentioned. Firstly, in contrast to factor analysis and cluster analysis, which are commonly used for dietary pattern analysis [27], the dietary indices typically summarize scores of the degree to which an individual’s diet conforms to specific dietary recommendations, i.e., dietary index is an “a priori” defined dietary pattern created on the basis of previous knowledge. Dietary indices can thus be fraught with uncertainties in selecting components to assign a score and subjectivity in defining cut-off points. In our analysis, all of the current dietary recommendations, on which CCDI constructed, were not especially relevant to puberty timing. Furthermore, dietary index does not consider the correlation structure of foods and nutrient intakes. It might be more applicable to focus on the variation in such nutrients, foods and eating behaviors that presumably affect the puberty timing. Finally, data deriving from a FFQ may not be representative of habitual dietary intake. However, our sensitivity analyses showed similar associations between diet quality deriving from 24-h recalls and puberty timing, which has partly overcome this potential limitation.

In conclusion, our data suggested that children with higher diet quality in prepuberty entered their puberty at a later age. This association was independent of pre-pubertal body fat.

## Electronic supplementary material

Below is the link to the electronic supplementary material.

Supplementary file1 (DOCX 54 KB)

## Data Availability

Not applicable.
