# Supplementary material for: The overall diet quality in childhood is prospectively associated with the timing of puberty
Source: Eur J Nutr. 2020 Nov 2;60(5):2423–34. doi: 10.1007/s00394-020-02425-8 (PMC8275527; doi:10.1007/s00394-020-02425-8)
Supplement: Supplementary file 1 — Supplementary file1 (DOCX 54 KB) [file 394_2020_2425_MOESM1_ESM.docx]

**Online Supporting Material**

**TableS1**Dietary intake^a^ by groups of diet quality and the medians (25th percentile, 75th percentile) of CCDI sub-scores^b^

|  | **Diet quality according to CCDI score** | | |  | Percentage of participants meeting dietary recommendations |
| --- | --- | --- | --- | --- | --- |
|  | Lower  (57.3-84.2)^c^ | Moderate  (84.9-107.1)^c^ | Higher  (107.5-135.7)^c^ | Sub-scores of CCDI |  |
| **Girls** |  |  |  |  |  |
| Grains, g/d | 476 (408, 582) | 351 (258, 382) | 167 (110, 268) | 5.6 (3.2, 8.0) | 12.3% |
| Vegetables, g/d | 92 (84, 135) | 165 (127, 183) | 278 (261, 295) | 4.8 (3.1, 7.5) | 16.2% |
| Fruits, g/d | 52 (31, 70) | 123 (95, 145) | 171 (145, 198) | 7.2 (3.7, 10.0) | 38.3% |
| Dairy and dairy products, g/d | 65 (48, 98) | 223 (198, 258) | 293 (275, 367) | 8.1 (3.6, 10.0) | 38.4% |
| Soybeans and its products, g/d | 2.1 (0, 6.5) | 15 (8.7, 19) | 67 (42, 95) | 4.3 (1.9, 9.8) | 26.1% |
| Meats, g/d | 82 (18, 145) | 62 (31, 109) | 47 (36, 79) | 3.1 (0, 7.5) | 14.4% |
| Fishes and shrimps, g/d | 0 (0, 0) | 9 (5, 27) | 29 (18, 42) | 0 (0, 5.8) | 9.0% |
| Eggs, g/d | 0 (0, 0) | 19 (12, 28) | 54 (38, 91) | 2.5 (0, 8.2) | 12.8% |
| Drinking water, mL/d | 375 (250, 475) | 600 (500, 725) | 950 (900, 1150) | 5.2 (3.1, 8.2) | 17.1% |
| SSBs^d^, mL/d | 173 (139, 198) | 72 (31, 115) | 0 (0, 0) | 4.2 (1.8, 10.0) | 26.2% |
| Vitamin A, μgRE/d | 195 (173, 227) | 348 (281, 364) | 482 (437, 498) | 5.8 (3.7, 9.2) | 22.1% |
| Fatty acids^e^ | 1.4 (1.1, 1.8) | 2.3 (1.9, 2.7) | 3.2 (2.9, 3.9) | 10.0 (4.1, 10.0) | 56.0% |
| Dietary fiber, g/d | 5.5 (4.1, 6.9) | 8.9 (7.1, 11) | 13 (9.2, 14) | 4.2 (2.6, 5.3) | 11.9% |
| Diet variety (servings)^f^ | 5 (3, 9) | 8 (5, 12) | 10 (8, 15) | 6.0 (4.0, 9.0) | 22.5% |
| Breakfast and dinner^g^ | 6 (4, 8) | 8 (6, 10) | 9 (7, 12) | 9.0 (8.0, 10.0) | 39.5% |
| Energy balance^h^ | 0.7 (0.5, 2.1) | 0.9 (0.5, 1.9) | 1.2 (0.6, 1.8) | 8.2 (3.8, 9.8) | 23.3% |
|  |  |  |  |  |  |
|  | Lower  (47.5-76.8)^i^ | Moderate  (77.5-104.1)^i^ | Higher  (104.7-133.6)^i^ | Distribution of sub-scores | Percentage of participants meeting dietary recommendations |
| **Boys** |  |  |  |  |  |
| Grains, g/d | 521 (472, 548) | 436 (297, 510) | 201 (156, 337) | 2.8 (0, 6.3) | 8.8% |
| Vegetables, g/d | 81 (67, 110) | 136 (117, 159) | 246 (223, 259) | 4.6 (2.2, 6.7) | 10.8% |
| Fruits, g/d | 20 (12, 43) | 153 (125, 181) | 187 (158, 201) | 6.3 (1.1, 10.0) | 31.1% |
| Dairy and dairy products, g/d | 87(65, 103) | 262 (215, 306) | 357 (323, 384) | 8.5 (5.2, 10.0) | 40.8% |
| Soybeans and its products, g/d | 0 (0, 0) | 17 (6.5, 29) | 67 (39, 113) | 4.5 (0, 10.0) | 25.0% |
| Meats, g/d | 87 (69, 196) | 72 (38, 121) | 49 (33, 81) | 1.3 (0, 6.1) | 8.5% |
| Fishes and shrimps, g/d | 0 (0, 0) | 9 (3, 23) | 28 (17, 51) | 0 (0, 7.2) | 7.0% |
| Eggs, g/d | 0 (0, 0) | 22 (13, 36) | 68 (49, 102) | 3.6 (0, 10) | 25.3% |
| Drinking water, mL/d | 475 (325, 550) | 625 (500, 775) | 1050 (825, 1175) | 5.9 (3.8, 9.1) | 20.0% |
| SSBs^d^, mL/d | 173 (115, 241) | 78 (45, 112) | 0 (0, 11) | 4.3 (2.5, 10.0) | 26.0% |
| Vitamin A, μgRE/d | 178 (147, 193) | 283 (261, 323) | 418 (395, 472) | 5.0 (3.2, 8.8) | 19.1% |
| Fatty acids^e^ | 1.5 (1.3, 1.7) | 2.3 (1.8, 2.8) | 3.5 (3.1, 3.7) | 9.8 (5.1, 10.0) | 57.6% |
| Dietary fiber, g/d | 4.3 (3.3, 5.6) | 7.1 (5.8, 8.8) | 9.8 (9.1, 12) | 3.7 (2.2, 4.4) | 9.5% |
| Diet variety^f^ | 5 (4, 8) | 7 (5, 10) | 10 (7, 14) | 5.0 (3.0, 8.0) | 19.1% |
| Breakfast and dinner^g^ | 7 (4, 9) | 8 (5, 12) | 10 (5, 13) | 9.0 (6.0, 10.0) | 29.3% |
| Energy balance^h^ | 1.1 (0.4, 2.3) | 0.9 (0.3, 1.5) | 1.0 (0.5, 1.3) | 8.2 (3.4, 9.8) | 22.8% |

^a^ All nutritional data represent crude values per day, dietary data was assessed using 24-hour record at baseline

^b^Data are presented as medians (25^th^ percentile, 75^th^ percentile) in tertiles. CCDI, Chinese Children Dietary Index.

^c^ Values are min-max in tertiles in girls

^d^ SSBs, sugar-sweetened beverages. SSBs were defined as beverages with added sugar, such as lemonades, fruit drinks (diluted and sugar-sweetened fruit juices), ice teas and so on. Juices made from 100% fruit were not classified as SSBs.

^e^ Ratio of poly- and monounsaturated fatty acids to saturated fatty acids, derived from the HEI-2010[[51](#_ENREF_51)].

^f^ Daily consumption of at least one serving from each of the food groups (grains, vegetables, fruits, dairy/beans and meats/poultry/fishes/eggs) was necessary to count the diet variety.

^g^ Frequencies per week, having breakfast and dinner with parents or grandparents.

**TableS2**Association of diet quality in childhood with puberty timing^a^

|  | | **Diet quality according to CCDI score** | | |  |
| --- | --- | --- | --- | --- | --- |
|  | | Lower  (57.3-84.2)^b^ | Moderate  (84.9-107.1)^b^ | Higher  (107.5-135.7)^b^ | *p_trend_* ^c^ |
| **Girls** |  | |  |  |  |
| **Age at Tanner stage B2（n=1752）** | | |  |  |  |
| Unadjusted model: | 1 | | 0.91 (0.81, 0.97) | 0.87 (0.79, 0.93) | 0.04 |
| Model 2^d^: | 1 | | 0.90 (0.79, 0.98) | 0.86 (0.81, 0.95) | 0.03 |
| Final model^e^: | 1 | | 0.89 (0.80, 0.99) | 0.85 (0.80, 0.94) | 0.02 |
| **Age at menarche (n=1752)** | | |  |  |  |
| Unadjusted model: | | 1 | 0.92 (0.86, 1.02) | 0.89 (0.84, 0.98) | 0.04 |
| Model 2^d^: | | 1 | 0.90 (0.85, 0.98) | 0.88 (0.83, 0.97) | 0.02 |
| Final model^e^: | | 1 | 0.90 (0.83, 0.96) | 0.88 (0.83, 0.98) | 0.02 |
|  | |  |  |  |  |
| **Boys** | | Lower  (47.5-76.8)^f^ | Moderate  (77.5-104.1)^f^ | Higher  (104.7-133.6)^f^ | *p_trend_* ^c^ |
| **Age at Tanner stage G2（n=2231）** | | |  |  |  |
| Unadjusted model: | | 1 | 0.91 (0.85, 0.99) | 0.87 (0.79, 0.96) | 0.03 |
| Model 2^d^: | 1 | | 0.89 (0.81, 0.98) | 0.85 (0.79, 0.98) | 0.02 |
| Final model^e^: | | 1 | 0.89 (0.83, 0.98) | 0.84 (0.878, 0.97) | 0.02 |
| **Age at voice break (n=2231)** | | |  |  |  |
| Unadjusted model: | | 1 | 0.91 (0.84, 1.02) | 0.87 (0.78, 0.98) | 0.04 |
| Model 2^d^: | | 1 | 0.89 (0.83, 0.97) | 0.86 (0.79, 0.95) | 0.03 |
| Final model^e^: | | 1 | 0.89 (0.81, 0.96) | 0.85 (0.77, 0.95) | 0.03 |

^a^Values are models adjusted hazard ratios (95% CI), HR= hazard ratio; diet quality was assessed with dietary data using 24-hour record, according to CCDI (Chinese Children Dietary Index)

^b^Values are min-max in tertiles in girls

^c^Pfor trend across CCDI tertiles were performed by including CCDI ordinals as continuous variables

^d^ Adjusted for paternal education level and energy intake at baseline

^e^Additionally adjusted for percent body fat at baseline

^f^Values are min-max in tertiles in boys
